# Gas6 derived from cancer-associated fibroblasts promotes migration of Axl-expressing lung cancer cells during chemotherapy

**DOI:** 10.1038/s41598-017-10873-2

**Published:** 2017-09-06

**Authors:** Ryu Kanzaki, Hisamichi Naito, Kazuyoshi Kise, Kazuhiro Takara, Daisuke Eino, Masato Minami, Yasushi Shintani, Soichiro Funaki, Tomohiro Kawamura, Toru Kimura, Meinoshin Okumura, Nobuyuki Takakura

**Affiliations:** 1 Department of Signal Transduction, Research Institute for Microbial Diseases, Osaka University, Suita, Japan; 2 Department of General Thoracic Surgery, Osaka University Graduate School of Medicine, Suita, Japan

## Abstract

Alterations to the tumor stromal microenvironment induced by chemotherapy could influence the behavior of cancer cells. In the tumor stromal microenvironment, cancer-associated fibroblasts (CAFs) play an important role. Because the receptor tyrosine kinase Axl and its ligand Gas6 could be involved in promoting non-small cell lung cancer (NSCLC), we investigated the role of Gas6 secreted by CAFs during chemotherapy in NSCLC. In a murine model, we found that Gas6 expression by CAFs was upregulated following cisplatin treatment. Gas6 expression might be influenced by intratumoral hypoperfusion during chemotherapy, and it increased after serum starvation in a human lung CAF line, LCAF^hTERT^. Gas6 is associated with LCAF^hTERT^ cell growth. Recombinant Gas6 promoted H1299 migration, and conditioned medium (CM) from LCAF^hTERT^ cells activated Axl in H1299 cells and promoted migration. Silencing Gas6 in LCAF^hTERT^ reduced the Axl activation and H1299 cell migration induced by CM from LCAF^hTERT^. In clinical samples, stromal Gas6 expression increased after chemotherapy. Five-year disease-free survival rates for patients with tumor Axl- and stromal Gas6-positive tumors (n = 37) was significantly worse than for the double negative group (n = 12) (21.9% vs 51.3%, p = 0.04). Based on these findings, it is presumed that Gas6 derived from CAFs promotes migration of Axl-expressing lung cancer cells during chemotherapy and is involved in poor clinical outcome.

## Introduction

Lung cancer is a leading cause of cancer-related mortality in industrialized countries^1^. Conventional treatment options for non-small cell lung cancer (NSCLC) are surgery, radiotherapy, and chemotherapy^2^. Chemotherapy or chemoradiotherapy followed by surgery is considered a viable treatment option for locally-advanced NSCLC^3–5^.

Although chemotherapy has cytotoxic effects on cancer cells, it may also have undesirable secondary effects. Cancer cells can develop drug resistance and enhanced aggressiveness during chemotherapy^6, 7^. It is reported that both phenomena are influenced by the tumor stromal microenvironment^8^ in which cancer-associated fibroblasts (CAFs) in particular play an important role^9^. We previously reported that CAFs can induce epithelial–mesenchymal transition (EMT), stemness and drug resistance in cancer cells^10–13^. Recently, alterations of the tumor stromal microenvironment due to chemotherapy have attracted considerable attention, in particular in lung cancer^14, 15^ where such alterations have become a matter of importance.

Axl, a member of the TAM family of receptor tyrosine kinases (RTKs), consisting of Tyro 3, Mer, and Axl^16^, may be a potential therapeutic target for NSCLC. Axl was originally identified in chronic myeloid leukemia cells and shown to transform normal cells^17^. It contributes to development and promotion not only of hematological malignancies but also solid tumors including NSCLC^18–20^. Thus, it was reported that Axl expression levels in clinical samples of NSCLC were associated with tumor progression and patient survival^21^. Gas6 is a natural ligand of TAM receptors, and binds with high affinity to Axl, causing its phosphorylation and activation of the signaling pathways^19^. Sources of Gas6 are considered to be cancer cells themselves and/or the tumor stromal microenvironment. Using mouse cancer models, two groups have shown that Gas6 produced by tumor stromal cells promotes solid tumor growth and drug resistance in leukemia^22, 23^. However, whether CAFs in human lung cancers could be a source of Gas6 remains unclear.

In the present study, we analyzed Gas6 expression in CAFs and its alteration by chemotherapy using a mouse model and cells derived from human lung cancers; we also examined the effects of Gas6 secreted by CAFs on lung cancer cells. Ultimately, we assessed the relationships among tumor Axl expression, stromal Gas6 and prognosis using clinical data.

## Results

### Gas6 expression in CAFs increases after CDDP treatment

**Figure 1:**
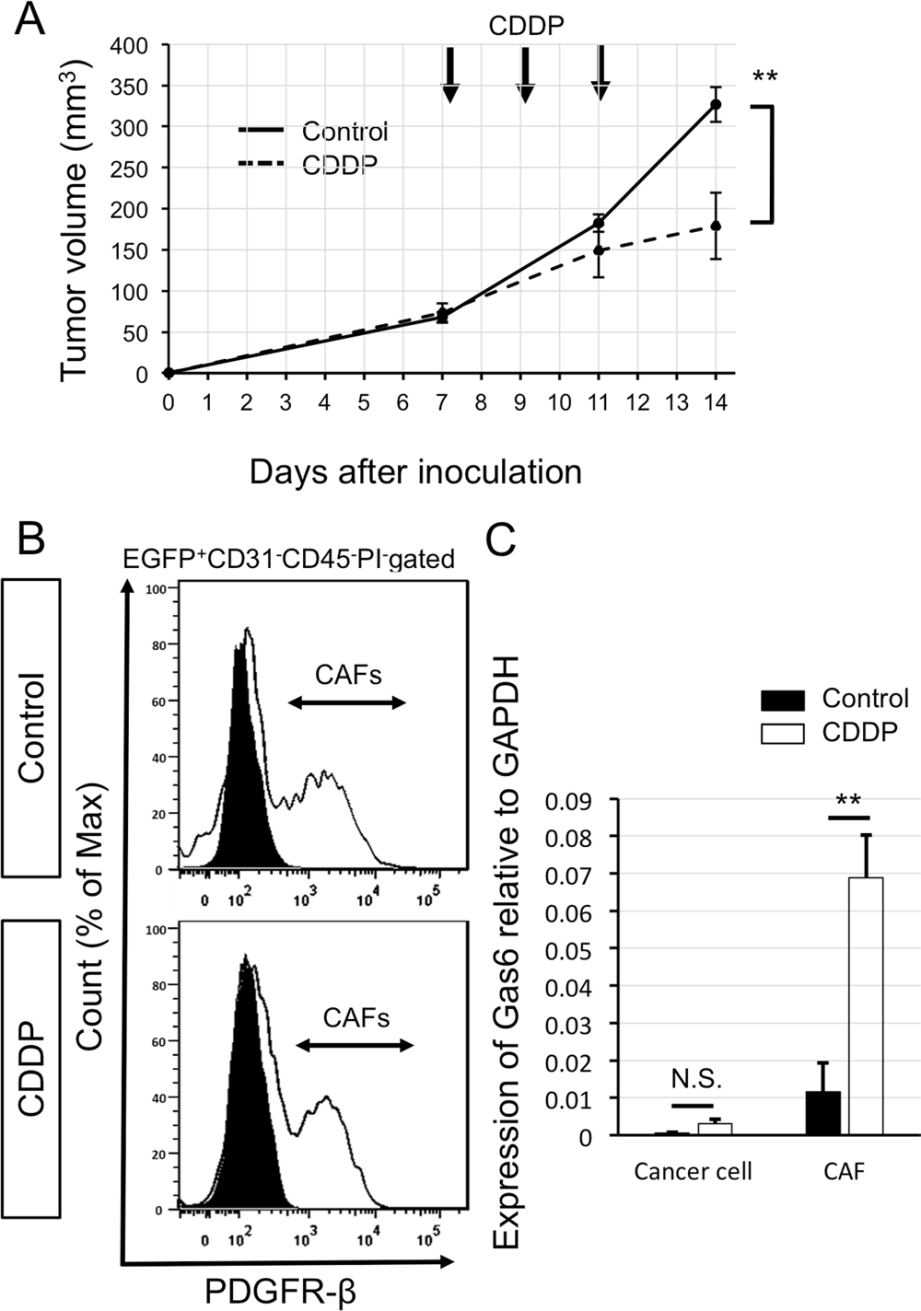
Gas6 expression in CAFs after cisplatin (CDDP) treatment. (**A**) Time course of tumor volume changes after CDDP administration *in vivo*. CDDP was administered to tumor-bearing mice inoculated with LLC cells on days 7, 9, and 11 as indicated by the arrows. Data show the means ± SEM (*n* = 6). **P < 0.01. (**B**) Representative flow cytometric analysis of cells obtained from tumor tissues on day 14. Upper panel: control group; lower panel: CDDP group. Black areas represent the isotype control. EGFP^+^CD31^−^CD45^−^PDGFR-β^+^ cells as shown in (**B**) were designated as CAFs. (**C**) qRT-PCR analysis of Gas6 expression in cancer cells and CAFs with or without CDDP treatment. Data show the mean ± SEM (n = 3); **p < 0.01.

We hypothesized that Gas6 expression in CAFs was altered by chemotherapy. We used a syngeneic mouse subcutaneous tumor model and PDGFR-β, which is expressed by vessel-associated pericytes and fibroblasts^24, 25^, as a marker for CAFs. Because Lewis lung carcinoma (LLC), a murine lung carcinoma cell line, expresses PDGFR-β (data not shown), we used EGFP mice to distinguish host-derived cells (EGFP^+^) from cancer cells (EGFP^−^). LLC cells were inoculated into EGFP mice, which were then treated with cisplatin (CDDP) (arrows, Fig. 1A). On day 14 after inoculation of LLC cells, tumors were dissected and cancer cells (EGFP^−^ cells) and CAFs (EGFP^+^ CD31^−^CD45^−^ PDGFR-β^+^ cells) were sorted (Fig. 1B). *Gas6* expression was not observed in cancer cells and this was not altered by CDDP treatment. However, *Gas6* expression in CAFs was markedly increased by CDDP treatment (Fig. 1C).

### Gas6 expression increases after serum starvation in human lung CAFs isolated from surgical specimens and Gas6 is associated with cell growth of CAFs

**Figure 2:**
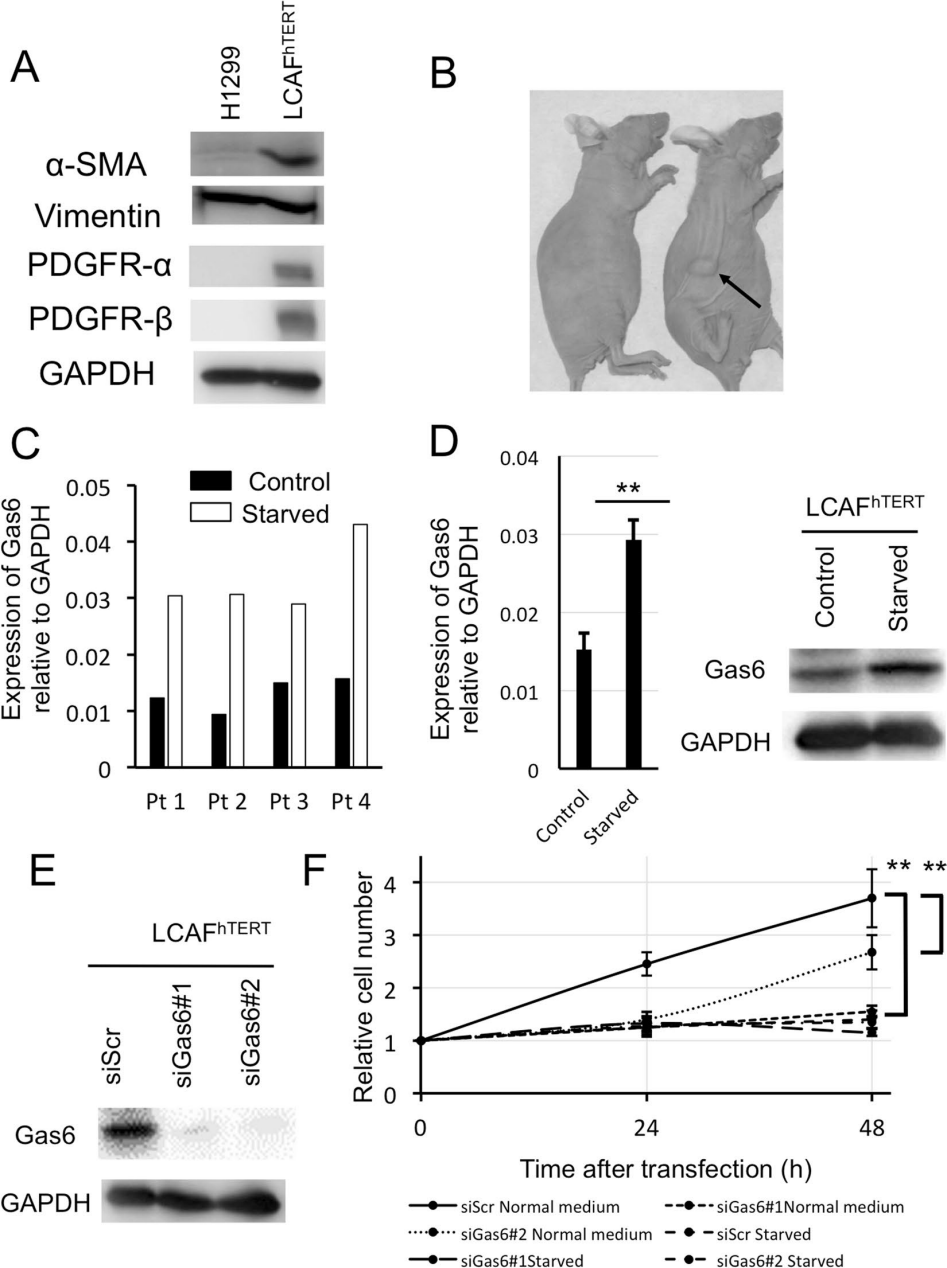
Gas6 expression in human lung CAFs isolated from surgical specimens. (**A**) Western blotting of αSMA, vimentin, PDGFR-α, PDGFR-β in LCAF^hTERT^ cells. H1299 cell lysates were used as the control. GAPDH was used as an internal control. (**B**) Tumorigenicity of H1299 is enhanced by LCAF^hTERT^. Images of tumors grown in mice inoculated with H1299 cells with or without LCAF^hTERT^. (**C**) qRT-PCR analysis of *Gas6* expression in primary CAFs from 4 patients (Pt) with or without serum starvation. (**D**) Left: qRT-PCR analysis of *Gas6* expression in LCAF^hTER^ cells with or without serum starvation. Data show mean ± SEM (n = 3). Right: Western blot of Gas6 expression in LCAF^hTERT^ with or without serum starvation. Serum starvation was performed by reducing FBS concentration to 1% in culture medium for 48 h. (**E**) Silencing of Gas6 in LCAF^hTERT^ by siRNA. Western blotting to assess Gas6 expression in LCAF^hTERT^ transfected with siRNAs. (**F**) Cell growth of LCAF^hTER^ cells transfected with siGas6 or scrambled siRNA (siScr) with normal medium (10% FBS) or serum-starved medium (1% FBS); **p < 0.01.

To study CAFs derived from human lung cancer, we isolated and cultured them from 5 patients and immortalized one of the cell lines (LCAF^hTERT^). This line had a typical spindle-shaped morphology and expressed CAF markers such as αSMA, vimentin, PDGFR-α, and PDGFR-β (Fig. 2A). To test whether LCAF^hTERT^ supports tumorigenicity of human lung cancer cells, we performed experiments coinoculating these cells together with human NSCLC H1299 cells into subcutaneous tissue in mice, using H1299 cells alone as a control. Six weeks after inoculation, H1299 coinoculated with LCAF^hTERT^ cells were found to have formed palpable tumors while H1299 cells alone did not do so. Thus, LCAF^hTERT^ supported tumorigenicity in this mouse subcutaneous tumor system (Fig. 2B).

Because we found that Gas6 expression was upregulated in CAFs by CDDP using a murine model, we hypothesized that Gas6 expression from CAFs could be regulated by CDDP exposure. Then we exposed LCAF^hTERT^ to different concentrations of CDDP *in vitro*, but Gas6 expression was not altered (data not shown). Next, we focused on blood perfusion in the tumor. Previous studies have reported that blood flow is reduced after chemotherapy in mouse tumor models and human cancers^26–30^ because of hypoperfusion due to the disruption of blood vessels. Therefore, we evaluated the influence of chemotherapy on apoptosis of endothelial cells in subcutaneous tumors (Supplementary Fig. 1A). The number of apoptotic blood vessels among all vessels tended to be higher in the CDDP-treated group than in the control group (Supplementary Fig. 1B), suggesting that decreased blood flow is associated with Gas6 upregulation in CAFs. Based on previous reports and on our findings, it is highly possible that Gas6 expression in CAFs is regulated by hypoperfusion. Because it has been reported that serum starvation could be used to mimic hypoperfusion inside the tumor^31, 32^, we performed serum starvation *in vitro* on CAFs derived from human lung cancers to investigate whether blood flow was associated with Gas6 upregulation in CAFs. Gas6 gene and protein were both upregulated after serum starvation in CAFs and in LCAF^hTERT^ cells (Fig. 2D). Next, we analyzed the effect of Gas6 on CAF growth. We silenced Gas6 expression in LCAF^hTERT^ by siRNA and observed cell growth *in vitro* (Fig. 2E). Silencing Gas6 significantly reduced LCAF^hTERT^ cell growth, which was also reduced by serum starvation. There was no significant difference between the cell number of LCAF^hTERT^ transfected with siGas6 and of that transfected with scrambled siRNA (Fig. 2F). These findings suggest that Gas6 is associated with CAF cell growth.

### Axl activation by Gas6 promotes migration of NSCLC cells

**Figure 3:**
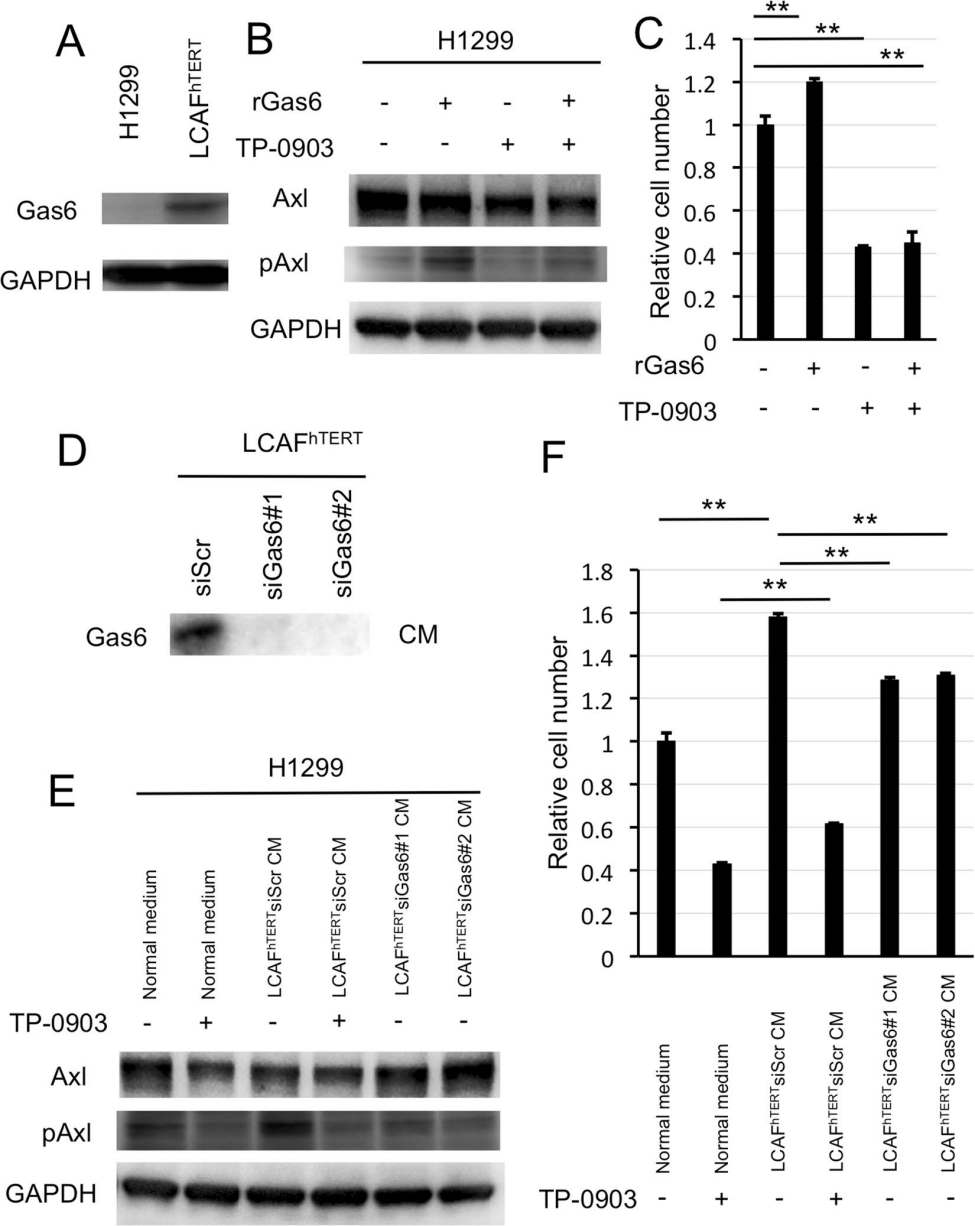
Migration of H1299 NSCLC cells enhanced by ligand-dependent Axl activation. (**A**) Western blotting to assess Gas6 expression in H1299 cells. Expression of Gas6 in LCAF^hTERT^ cells was used as a positive control. (**B**) Phosphorylation of Axl was analyzed by Western blotting of whole cell lysates using different antibodies. GAPDH was used as an internal control. H1299 cells were stimulated for 15 min with 400 nM recombinant human Gas6 (rGas6). H1299 cells were treated with or without TP-0903 (0.2 µmol/L) for 24 h. (**C**) Migration of H1299 cells was analyzed using rGas6 (400 nM) added to the lower chamber. H1299 cells were treated with or without TP-0903 (0.2 µmol/L) for 24 h. (**D**) Western blotting of conditioned medium from LCAF^hTERT^ transfected with siGas6 or siScr (control) to assess whether they contains Gas6 secreted by CAFs. (**E**) Phosphorylation of Axl in H1299 cells analyzed by Western blotting after stimulation with conditioned medium from siRNA-transfected LCAF^hTERT^. H1299 cells were treated with or without TP-0903 (0.2 µmol/L) for 24 h. The medium (DMEM containing 10% FBS) was used as control. (**F**) Migration of H1299 cells analyzed using conditioned medium of siRNA-transfected LCAF^hTERT^. H1299 cells were treated with or without TP-0903 (0.2 µmol/L) for 24 h. The medium (DMEM containing 10% FBS) was used as control. The relative number of migrated H1299 cells is indicated on the y-axis. Data show the mean ± SEM (n = 3); **p < 0.01.

To investigate the possible function of the stromal Gas6–tumor Axl axis, we stimulated NSCLC cells with Gas6 *in vitro*. From a panel of Axl-expressing NSCLC cell lines^19, 20, 33^, we selected H1299 because Ax1 had been reported to be further phosphorylated in these cells by exogenously added Gas6^20^. The expression of Gas6 in H1299 cells was barely detectable (Fig. 3A). The amount of activated (phosphorylated) Ax1 increased in a ligand-dependent manner. Inhibition of Axl by Axl-specific inhibitor TP-0903^34^ decreased Axl expression and inhibited phosphorylation of Axl by Gas6 (Fig. 3B). Because activation of Axl may be involved in cell migration^16^, we performed migration assays. Stimulation by recombinant Gas6 indeed promoted migration of H1299 cells, and inhibition of Axl by TP-0903 decreased migration. Under inhibition of Axl by TP-0903, stimulation by recombinant Gas6 did not promote cell migration (Fig. 3C). We then tested whether Gas6 secreted by CAFs had the same effect. We first silenced Gas6 expression in LCAF^hTERT^ by siRNA and confirmed that conditioned medium from LCAF^hTERT^ transfected with siGas6 did not contain Gas6 (Fig. 3D). We then found that conditioned medium (CM) from LCAF^hTERT^ transfected with siGas6 did not activate Axl in H1299 cells, whereas CM from LCAF^hTERT^ cells transfected with siScr did. Under inhibition of Axl by TP-0903, stimulation by CM from LCAF^hTERT^ cells transfected with siScr did not activate Axl (Fig. 3E). Next, we performed a migration assay with H1299 cells using CM from LCAF^hTERT^. The number of migrating cells induced by CM from LCAF^hTERT^ transfected with siScr was significantly higher than that induced from control cells (normal medium). The number of migrating cells induced by CM from LCAF^hTERT^ cells transfected with siGas6 was significantly lower than that induced by CM from LCAF^hTERT^ cells transfected with siScr, suggesting that the effect on migration by the CM from LCAF^hTERT^ cells can be partially explained by Gas6 contained in the CM. Inhibition of Axl by TP-0903 decreased the number of migrating cells compared with the control; however, even under inhibition of Axl by TP-0903, CM from LCAF^hTERT^ cells transfected with siScr induced cell migration (Fig. 3F). These data suggest that 1) Gas6 secreted by CAFs promotes migration of H1299 NSCLC cells, and 2) other factor(s) contained in the CM from CAFs may promote migration of H1299 NSCLC cells.

### Expression of Axl and Gas6 in clinical samples

**Figure 4:**
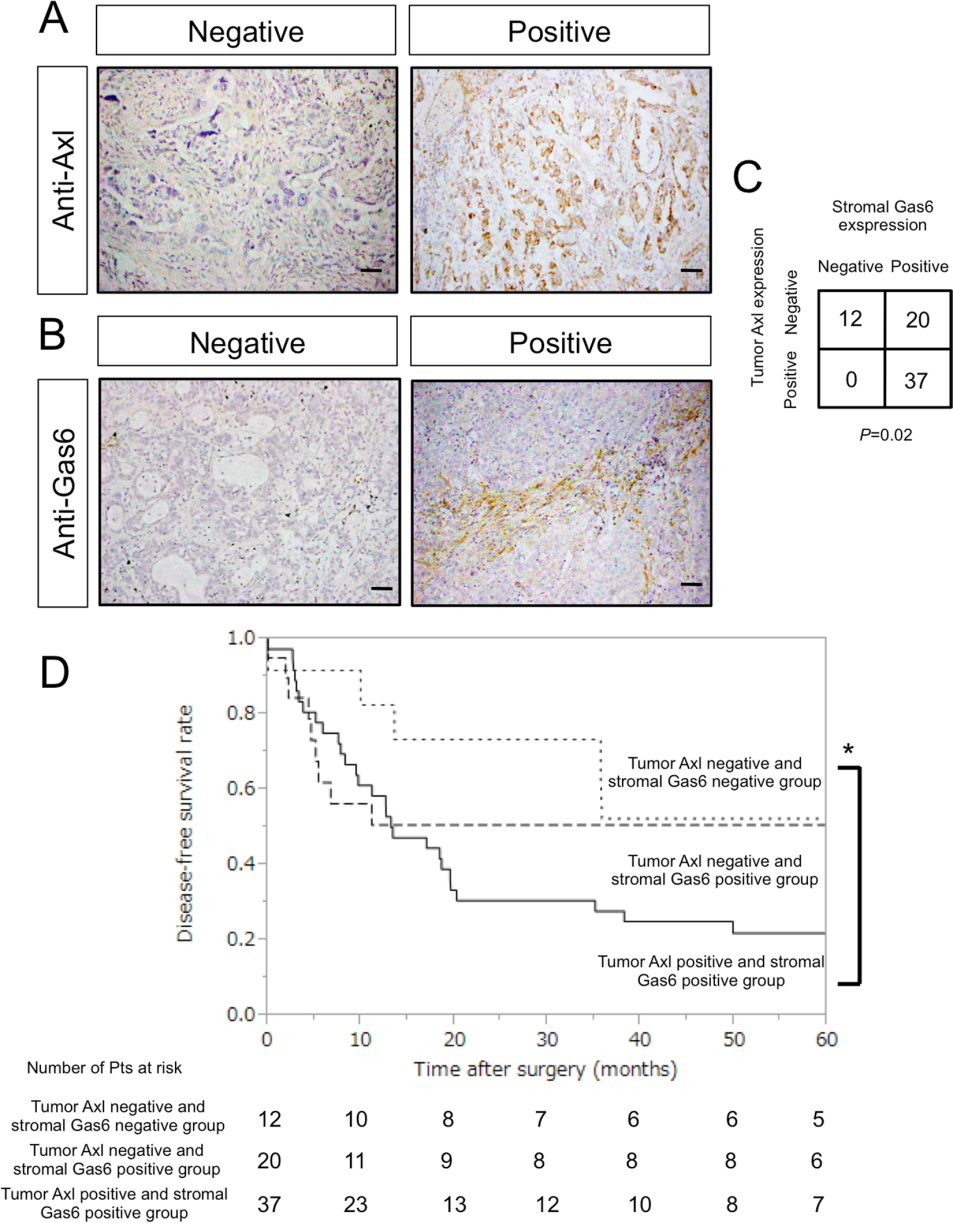
Expression of Axl and Gas6 in clinical samples. (**A,B**) Immunohistochemical analysis of Axl (**A**) or Gas6 (**B**) in non-small cell lung cancer tissues from patients who underwent surgery following preoperative chemotherapy or chemoradiotherapy. Insets show higher magnification of the areas indicated in the boxes. Scale bar, 50 μm. (**A**) Representative images showing tumor Axl-negative tumor tissues (left), and tumor Axl-positive tumor tissues (right). Tissues were stained with an anti-Axl antibody (brown) and counterstained with hematoxylin. (**B**) Representative images showing stromal Gas6-negative tumor tissues (left) and stromal Gas6-positive tumor tissues (right). Tissues were stained with an anti-Gas6 antibody (brown) and counterstained with hematoxylin. (**C**) Correlation between tumor Axl expression and stromal Gas6 expression in tumor tissues observed in (**A and B**). (**D**) Correlation between tumor Axl, stromal Gas6 expression and survival. Kaplan–Meier plot of disease-free survival in patients with non-small cell lung cancer who underwent surgery following preoperative chemotherapy or chemoradiotherapy, stratified according to tumor Axl and stromal Gas6 expression. Five-year disease-free survival in the patients with tumors expressing both tumor Axl and stromal Gas6 (n = 37) was significantly worse than in the both-negative group (n = 12) (21.9% vs 51.3%, p = 0.04). The five-year disease-free survival of tumor Axl-negative and stromal Gas6-positive group was 50.7%, and the difference in survival between this group and both-positive or both-negative group was not significant (p = 0.20 and 0.49, respectively); *p < 0.05.

Characteristics of 69 patients studied are shown in Table 1. Following identification of tumor and stromal compartments by hematoxylin-eosin (HE) staining, tumor Axl and stromal Gas6 expression was determined by immunohistochemistry. In a fraction of patients, Axl was found to be expressed in the tumor and Gas6 in both tumor and stroma (Fig. 4A and B). TumorAxl was expressed in 37 (53%) of patients’ tumors but not in the remaining 32 (47%). Stromal Gas6 expression was seen in 57 (83%) of patients, and was lacking in 12 (17%). Relationships between tumor Axl expression and stromal Gas6 expression are shown in Fig. 4C. All tumors expressing tumor Axl also expressed stromal Gas6 (p = 0.02).

**Table 1:** Patients’ characteristics.

Characteristics	Number of patients
Age (mean ± SD)	59.9 ± 10.0
Sex (male/female)	12/57
Preoperative therapy (CRT/CT)	33/36
Regimen of preoperative chemotherapy	
CDDP + VDS	11
CDDP + VNR	10
CDDP + DTX	8
CDDP + VDS + MMC	6
CDDP + ETP	1
CDDP + PTX	1
CBDCA + PTX	18
CBDCA + DTX + GEM	8
CBDCA + ETP	5
CBDCA	1
Pathologic stage	
IA	8
IB	11
IIA	5
IIB	13
IIIA	21
IIIB	5
IV	6
Histologic type	
Adenocarcinoma	34
Squamous cell carcinoma	29
Other histologic types	6

CBDCA, carboplatin; CDDP, cisplatin; CRT, chemoradiotherapy; CT, chemotherapy.

DTX, docetaxel; ETP, etoposide; GEM, gemcitabine; MMC, mitomycin C; PTX, pacritaxel; SD, standard deviation; VDS. vindesine; VNR, vinorelbine.

Changes in stromal Gas6 expression induced by chemotherapy were assessed in 22 patients with biopsy samples obtained before chemotherapy. Stromal Gas6 was negative before chemotherapy and remained negative in 3 (14%) patients, was negative before chemotherapy and changed to positive in 11 (50%) patients, and was positive before chemotherapy and remained positive in 8 (36%) patients. Overall, stromal Gas6 expression tended to increase after chemotherapy. These data are compatible with our experimental data shown in Fig. 1. Next, we analyzed the relationship between tumor Axl and stromal Gas6 expression, and prognosis. Five-year disease-free survival in patients with tumors expressing both tumor Axl and stromal Gas6 (n = 37) was significantly worse than in the both-negative group (n = 12) (21.9% vs 51.3%, p = 0.04) (Fig. 4D). The five-year disease-free survival of the tumor Axl-negative and stromal Gas6-positive group was 50.7%, and the difference in survival between this group and both-positive group or both-negative group was not significant (p = 0.20 and 0.49, respectively).

Recurrences in the 29 patients with tumors expressing tumor Axl and stromal Gas6 after surgery following preoperative chemotherapy or chemoradiotherapy were as follows: distant metastasis in 21 (brain in 10, lung in 7, bone in 2, adrenal gland in 1, skin in 1), local recurrence in 7, and unknown in the remaining patient. Distant metastases were detected in 72% of the patients. These results are in accordance with the paracrine effect of Gas6 expressed and secreted by CAFs in promoting migration of NSCLC cells, shown in Fig. 3.

## Discussion

In this study, we demonstrated that Gas6 expression by CAFs increases during chemotherapy and that its secretion by CAFs promotes proliferation and migration of lung cancer cells. Furthermore, we confirmed an increase in stromal Gas6 expression during chemotherapy using clinical samples and showed that tumor Axl and stromal Gas6 expression are associated with poor prognosis.

CAFs play an important role in the tumor stromal microenvironment^9^. We previously reported that CM from fibroblasts induces EMT and stem cell-like characteristics in NSCLC cells^11^. We have also investigated the roles of secreted factors and cell surface molecules expressed by CAFs in supporting cancer cells. We previously demonstrated that lung cancer cells and fibroblast cells interact through TGF-β and IL-6 pathways; IL-6 enhances EMT and tumor progression by stimulating TGF-β signaling^10^. On the other hand, using CAFs derived from CD44-deficient mice, we demonstrated that CD44 expressed on CAFs induces stemness and drug resistance in cancer cells^13^. Recently, research on alterations to the tumor stromal microenvironment by treatment-induced damage has attracted particular attention in the context of overcoming drug resistance and cancer malignancy. Sun and colleagues reported that increased expression of WNT16B in fibroblasts caused by treatment-associated DNA damage responses promotes EMT in neoplastic prostate epithelium through paracrine signaling^14^. Lotti and colleagues reported that chemotherapy-treated CAFs promoted self-renewal of cancer-initiating cells and enhanced tumor growth through increased expression of IL-17A^15^. Therefore, we focused on the alteration of the tumor stromal microenvironment by chemotherapy in lung cancer.

Axl is frequently overexpressed and phosphorylated in lung cancer. Rikova and colleagues conducted a proteomic study on phosphorylated RTKs in human lung cancer cell lines and clinical samples. In their report, Axl is ranked among the top 20 phosphorylated RTKs in human lung cancer cell lines and clinical samples^33^. Activating mutations in Axl have not been reported and activation of Axl occurs in either a ligand-independent or -dependent autocrine/paracrine manner^16^. Previous *in vitro* studies demonstrated that ligand-independent receptor dimerization and activation can occur when Axl is overexpressed^35, 36^. Axl was highly phosphorylated under normal culture conditions and did not respond further to exogenous Gas6 in A549 cells^19, 20^. In addition to ligand-independent activation, Axl can also be activated in an autocrine/paracrine manner in humans because Gas6 is frequently overexpressed in lung cancer cells and is found in the plasma^21, 37^. In the present study, we demonstrated the presence of Gas6 in CAFs isolated from human lung cancer. To the best of our knowledge, our study is the first to report CAFs as a source of Gas6.

Several studies suggest that secretion of Gas6 from the tumor stromal microenvironment occurs in mice. Using a subcutaneous tumor model, Loges and colleagues^22^ demonstrated that Gas6 is produced by tumor-infiltrating leukocytes. They reported that circulating leukocytes produce minimal Gas6 but once they have infiltrated into the tumor, they upregulate Gas6 expression, contributing to tumor growth. Ben-Batalla and colleagues^23^ reported that Gas6 was expressed by bone marrow–derived stromal cells (BMDSCs). They showed that acute myeloid leukemia cells induce expression and secretion of Gas6 by BMDSCs, and Gas6 in turn mediates proliferation, survival, and chemo-resistance of the Axl-expressing acute myeloid lymphoma cells. However, secretion of Gas6 in the tumor stromal microenvironment in humans has not been previously reported. Furthermore, we demonstrated that the expression of Gas6 in CAFs increases after serum starvation (Fig. 2C,D), consistent with similar observations in NIH3T3 murine fibroblasts^38^. Gas6 is associated with CAF cell growth (Fig. 2F). We believe that CAFs strive to survive during chemotherapy.

In the present study, we found a positive relationship between tumor Axl expression and stromal Gas6 expression (Fig. 4C) and an increase in stromal Gas6 expression after chemotherapy. Furthermore, patients with tumors expressing tumor Axl and stromal Gas6 had poorer survival (Fig. 4D). Although a relationship between Axl expression and prognosis had been reported in NSCLC patients without preoperative therapy^21^, there were no data on relationships between Axl expression and prognosis using specimens from patients receiving preoperative therapy followed by surgery. The positive relationship between tumor Axl expression and stromal Gas6 expression remains unexplained. Ye and colleagues developed an anti-Axl monoclonal antibody^39^ which blocks Axl function not only by inhibiting the binding of Gas6 but also by downregulating Ax1 expression. Therefore, it is possible that Axl expression is upregulated by Gas6 binding in a positive feedback circuit; however, further investigation is required to clarify this issue.

This study has some limitations. Firstly, we could not produce a reliable experimental model using primary culture of CAFs from a syngeneic mouse subcutaneous tumor model. The contamination from a modicum of LLC cancer cells into sorted CAFs could not be completely prevented. Because these contaminated cancer cells proliferate very rapidly compared with CAFs, experiments using CM of primary sorted CAFs could not be conducted. Therefore, we used immortalized human CAFs for further *in vitro* experiments. Secondly, the precise mechanism of regulation of Gas6 expression during chemotherapy has not been completely explained. We hypothesized that Gas6 expression from CAF was regulated by CDDP exposure; however, Gas6 expression was not altered by direct exposure to CDDP *in vitro*. On the other hand, previous reports demonstrated that vascularity in tumors can be reduced by chemotherapy in experimental and clinical settings. In a mouse subcutaneous tumor model, apoptosis of tumor vascular endothelial cells was induced^29^, and significantly decreased blood perfusion was observed after chemotherapy^26^. Decreased blood flow due to chemotherapy has been also observed in clinical settings by positron emission tomography, perfusion CT, and diffuse optical monitoring^27, 28, 30^. In addition to these findings, we demonstrated the presence of increased apoptotic blood vessels after CDDP treatment (Supplementary Fig. 1). Based on these findings, we believe that intratumoral hypoperfusion can explain Gas6 upregulation in CAFs; however, further studies are required on this issue.

We have demonstrated that one pathway by which an altered tumor stromal microenvironment following chemotherapy promotes malignancy of lung cancer cells is through the Gas6–Axl axis. Combination chemotherapy with small molecules or antibodies against Axl or Gas6 may therefore represent a new therapeutic option in the future^39, 40^.

## Materials and Methods

All the experiments were performed in accordance with the institutional guidelines and regulations. Experiments using the tissue samples from patients were performed following approval of the Ethical Review Board for Clinical Studies at Osaka University, and written informed consent was obtained from all the patients.

### Cell culture

H1299 cells were purchased from the American Type Culture Collection (ATCC, Manassas, VA, USA) and maintained in RPMI-1640 with 10% fetal bovine serum (FBS; Sigma-Aldrich St Louis, MO, USA) and penicillin/streptomycin (Life Technologies, Carlsbad, CA, USA). Lewis lung carcinoma cells (LLC) were purchased from the Riken cell bank (Tsukuba, Japan) and maintained in Dulbecco’s modified Eagle’s medium (DMEM) (Sigma-Aldrich) with 10% FBS and penicillin/streptomycin.

### Mice, subcutaneous tumor model and cisplatin administration

All experiments were performed in accordance with the guidelines of the Osaka University Committee for animal experiments. C57BL/6-Tg (CAG-EGFP) male or female mice (EGFP mice, 7–8 weeks of age), C57BL/6 female mice (7–8 weeks of age), and KSN female mice (7–8 weeks of age) were purchased from Japan SLC (Shizuoka, Japan). Subcutaneous inoculation was performed by injecting 10^6^ LLC cells into the flanks of the mice. For coinoculations, 1 × 10^5^ LCAF^hTERT^ cells and 1 × 10^6^ H1299 cells were injected. Tumor volumes were measured with calipers and calculated as width × width × length × 0.52. On day 7, 9, and 11 after subcutaneous inoculation, mice were treated with intraperitoneal injections of 5 mg/kg body weight cisplatin (Bristol-Myers, Tokyo, Japan). Flow cytometric analysis and immunohistochemistry was performed on day 14 after inoculation.

### Quantitative polymerase chain reaction

Total RNA was isolated using RNAeasy Kits (Qiagen, Valencia, CA, USA) according to the manufacturer’s instructions. RNA was reverse transcribed using the ExScript RT Reagent Kit (Takara, Kyoto, Japan). Quantitative reverse-transcription polymerase chain reaction (qRT-PCR) was performed using SYBR Premix Ex Taq II (Takara) on an Mx3000 system (Stratagene, La Jolla, CA, USA). Levels of the specific amplified cDNAs were normalized to the levels of the glyceraldehyde-3-phosphate dehydrogenase (GAPDH) housekeeping control cDNA. We used the following primer sets: 5′-TGG CAA AGT GGA GAT TGT TGC C-3′ and 5′-AAG ATG GTGATG GGC TTC CCG-3 for murine *GAPDH*, 5′-CCC CCG TGA TTA GAC TAC GC-3′ and 5′-ATC CAG GTG CTG TCT GAA CG-3′ for murine *Gas6*, 5′-GAG TCA ACG GAT TTG GTC GT-3′ and 5′-TGG AAG ATG GTG ATG GGA TT-3 for human *GAPDH*, and 5′-GGA CAT GGA CAC CTG TGA GG-3′ and 5′-GGC CCA GGT ACA AGG ACT TC-3′ for human *Gas6*.

### Western blotting

Recombinant human Gas6 (885-GS, R & D Systems, Minneapolis, MN, USA) was used to stimulate H1299 cells. For detecting phosphorylation, cell extracts were prepared by lysing the cells in buffer containing 1 mM EDTA, 5 mM NaF, 0.5 mM sodium orthovanadate, 1 mM dithiothreitol (DTT), and 0.1 mM phenylmethylsulfonyl fluoride (PMSF), and protein inhibitor cocktail (Nacalai Tesque, Kyoto, Japan). Lysates from whole cells were separated by 7.5% or 10%SDS-PAGE. Proteins were then transferred onto nylon membranes (Amersham, Buckinghamshire, UK) and incubated with mouse anti-α-SMA antibody (clone 1A4, Sigma-Aldrich), mouse anti-vimentin antibody (vim3B4, Dako), rabbit anti-PDGFR-α antibody (#3164, Cell Signaling Technology), rabbit anti-PDGFR-β antibody (#3169, Cell Signaling Technology), goat anti-Axl antibody (AF154, R&D Systems), rabbit anti-phospho Axl antibody (AF2228, R&D Systems), goat anti-Gas6 antibody (AF885, R&D Systems) or mouse anti-GAPDH antibody (Chemicon, Temecula, CA, USA) according to the manufacturers’ protocols. Proteins were detected with horseradish peroxidase-conjugated anti-rabbit, anti-goat, or anti-mouse secondary antibodies (Jackson ImmunoResearch, West Grove, PA, USA) and ECL reagents (GE Healthcare, Milwaukee, WI, USA). The blots were scanned using an imaging densitometer LAS-3000 mini (Fujifilm, Tokyo, Japan).

### Flow cytometric analysis and cell sorting

Single-cell suspensions from tumors were prepared using a standard protocol^13, 41^. Fluorescence-activated flow cytometry and cell sorting (FACS) were performed on a FACSAria (BD Biosciences, San Diego, CA, USA) as described previously^13^. The antibodies used for flow cytometry were APC-conjugated rat anti-CD31 and anti-CD45 antibodies (BD Biosciences), and biotinylated rat anti-PDGFR-β antibody (clone APB5, eBioscience, San Diego, CA, USA). Following incubation with streptavidin PE (BD Biosciences), dead cells were stained with propidium iodide (Sigma-Aldrich, St Louis, MO, USA).

### Immunofluorescence of mouse tumor sections

Fixed specimens were embedded in OCT compound (Sakura Finetek, Tokyo, Japan) and sectioned (10-μm thick slices). For immunofluorescence analyses, rat anti-CD31 antibody (1/200) (BD Biosciences, San Diego, CA, USA) was used to stain vascular endothelial cells and rabbit anti-cleaved caspase-3 antibody (1/200) (#9579, Cell Signaling Technology, Danvers, MA, USA) to stain apoptotic cells. Anti-rat IgG Alexa Fluor 488 and anti-rabbit IgG Alexa Fluor 546 (Invitrogen, Carlsbad, CA, USA) were used as secondary antibodies. Samples were visualized using a Leica DM5500B and processed with the Leica application suite (Leica Microsystems, Bensheim, Germany). All images shown are representative of more than 3 independent experiments. Vessels and apoptotic cells were counted in more than 3 different fields in each tumor at a magnification of ×100.

### Isolation and primary culture of human lung CAFs

Human lung cancer tissues obtained by pulmonary resection were placed in Dulbecco’s modified Eagle’s medium (DMEM) (Sigma-Aldrich St Louis, MO) supplemented with antibiotics for immediate transportation on ice to the laboratory. Tissues were minced into small pieces and digested for 1 h at 37 °C in HBSS (Life Technologies, Carlsbad, CA, USA) containing 2 mg/mL collagenase A (Roche Diagnostics, Mannheim, Germany). The cell suspension was filtered first through a stainless steel wire mesh (500 µm) and then a 100-µm cell strainer (BD Biosciences). Cells in DMEM containing 10% FBS were plated into 100-mm tissue culture plates. Primary cells used in the experiments were not cultured beyond 10 passages. The tissue samples were used following approval of the Ethical Review Board for Clinical Studies at Osaka University (control number 10257-2). Written informed consent was obtained from all the patients.

### Immortalization of human lung CAFs by human telomerase

To facilitate the *in vitro* studies of human lung CAFs (LCAFs), primary cultured LCAFs were immortalized using a human telomerase (hTERT)-expressing lentivirus^42, 43^. LCAFs at an early passage were infected with the lentivirus (kindly provided by Dr R.A. Weinberg) and selected with neomycin. The resultant cell line was designated LCAF^hTERT^.

### siRNA transfection

Gas6 expression in LCAF^hTERT^ was transiently knocked down with small interfering RNAs (siRNAs). Lipofectamine RNAiMAX reagent (Invitrogen) was used for the siRNA transfection, following the manufacturer’s protocols, and experiments were done 24 h after transfection. We used 3 different siRNA oligonucleotides specific for Gas6 purchased from Thermo Fisher Scientific (Waltham, MA, USA).

### Inhibition of Axl

H1299 cells were treated with an Axl-specific inhibitor TP-0903 (Selleck Chemicals LLC, Houston, TX, USA) for 24 h (0.2 µmol/L)^34^.

### Cell growth curve analysis

LCAF^hTERT^ cells were uniformly seeded (2 × 10^4^/well) in triplicates into 24-well dishes. After 24 h, the cells were transfected with siRNA (siScr or siGas6). After 24 h, the medium was removed and replaced by 0.5 mL of fresh normal medium (DMEM containing 10% FBS) or medium for serum starved condition (DMEM containing 1% FBS). After 24 or 48 h, cells were counted using a hemocytometer.

### Migration assay

The migration assay was performed using Transwells (pore size, 8 μm, #3422, Costar, New York, NY, USA) in 24-well dishes. Twenty-four hours after transfection with siRNA of LCAF^hTERT^, cells were starved of serum starvation by reducing the FBS concentration to 1% in the culture medium for 48 h. Conditioned medium was then collected and 10% FBS was added just before the migration assay. A total of 2 × 10^4^ cells in 100 μL of serum-free medium was placed in the upper chamber, and 600 μL of the conditioned medium prepared as described above were placed in the lower chamber. After 6 h of incubation, cells were fixed in 4% PFA for 30 min and stained with Hoechst 33342 (5 μg/mL) (Sigma-Aldrich). Cells on the upper membrane surface were removed with a cotton swab. Cells on the lower side of the filters were counted under a fluorescence microscope. Each group was plated in triplicate in each experiment, and each experiment was repeated at least 3 times.

### Study population

Between 1996 and 2011, 86 patients with NSCLC underwent surgery following preoperative chemotherapy or chemoradiotherapy at the Osaka University Hospital. The present study includes patients who underwent induction therapy followed by surgery and patients who underwent salvage surgery following a definitive chemotherapy or chemoradiotherapy with good response. Indications for intended surgery following induction therapy or salvage surgery in our hospital were previously described^3^. One patient died perioperatively and was excluded from the analysis. Diagnosis of NSCLC and examination of pathologic response was performed by pathologists in our hospital. Of 85 patients, 16 (19%) achieved a pathologic complete response. The latter were excluded from subsequent immunohistochemistry studies and survival analysis because their cancer tissues were inappropriate for immunohistochemical evaluation of tumor cells. Thus, a total of 69 patients was analyzed. Thirty-seven patients underwent cisplatin (CDDP)-based chemotherapy and the remaining 32 received carboplatin (CBDCA)-based chemotherapy. Two cycles of chemotherapy were generally performed in patients who underwent intended induction therapy, while 2 to 9 cycles of chemotherapy were given to patients who underwent salvage surgery. In patients who received preoperative chemoradiotherapy, irradiation was generally concurrent with chemotherapy and limited to 40 Gy, with few exceptions. Staging was assessed according to the general rules for clinical and pathological recording of lung cancer from the Japan Lung Cancer Society^44^. Biopsy samples obtained before administration of preoperative chemotherapy were available for 22 of the 69 patients. These samples were obtained by transbronchial biopsy in 14 patients, computed tomography (CT)-guided needle biopsy in 7, and mediastinoscopy in one. The median follow-up period was 161 months (range 47–227). Specimens were examined following approval of the Ethical Review Board for Clinical Studies of Osaka University (control number 10026-3). Written informed consent was obtained from all the patients.

### Immunohistochemistry of clinical samples

Immunohistochemistry was performed as described previously^5^. Briefly, paraffin-embedded sections (2 µm thick) were prepared, deparaffinized, rehydrated, and subjected to antigen retrieval for 10 min at 121 °C in citrate buffer at pH 6 (S1699, Dako, Glostrup, Denmark). The sections were first incubated at 4 °C overnight with the primary antibodies goat anti-Axl (1/40) (AF154, R & D systems, Minneapolis, MN, USA) or goat anti-Gas6 (1/100) (AF885, R&D Systems), and thereafter with a secondary biotinylated rabbit anti-goat IgG (1/200) (Chemicon, Temecula, CA, USA); the signal was developed using ABC kits (Vector Laboratories, Burlingame, CA, USA). For the visualization of HRP, diaminobenzidine (Dojindo, Kumamoto, Japan) was used. Sections were counter-stained with hematoxylin. Tumor cells were identified by hematoxylin staining. Following identification of tumor and stromal compartments by hematoxylin-eosin (HE) staining, tumor Axl and stromal Gas6 expression was determined by immunohistochemistry. Tumor Axl expression was categorized as negative or positive, as was stromal Gas 6 expression.

### Statistical analysis

Statistical analysis was performed using JMP software (SAS Institute, Cary, NC, USA). All data are presented as means ± SEM. When two groups were compared, the two-sided Student’s *t*-test was used. Survival after surgery was calculated using the Kaplan–Meier method, and statistical significance was determined by the log-rank test. A p value < 0.05 was considered statistically significant.

## Electronic supplementary material

Supplementary Figure 1
