# Supplementary material for: Gas6 derived from cancer-associated fibroblasts promotes migration of Axl-expressing lung cancer cells during chemotherapy
Source: Sci Rep. 2017 Sep 6;7:10613. doi: 10.1038/s41598-017-10873-2 (PMC5587707; doi:10.1038/s41598-017-10873-2)
Supplement: Supplementary file 1 — Supplementary Figure 1 [file 41598_2017_10873_MOESM1_ESM.pdf]

Gas6 derived from cancer-associated fibroblasts promotes migration of Axl-expressing lung cancer cells during chemotherapy

Ryu Kanzaki[a,b], MD, Hisamichi Naito[a], MD, PhD, Kazuyoshi Kise[a], MD, PhD, Kazuhiro Takara[a], Daisuke Eino[a], MD, Masato Minami [b], MD, PhD, Yasushi Shintani[b], MD, PhD, Soichiro Funaki[b], MD, PhD, Tomohiro Kawamura[b], MD, PhD, Toru Kimura[b], MD, PhD, Meinoshin Okumura[b], MD, PhD, Nobuyuki Takakura[a], MD, PhD

[a] Department of Signal Transduction, Research Institute for Microbial Diseases, Osaka University, Suita, Japan.

[b] Department of General Thoracic Surgery, Osaka University Graduate School of Medicine, Suita, Japan.

Supplementary Figure 1

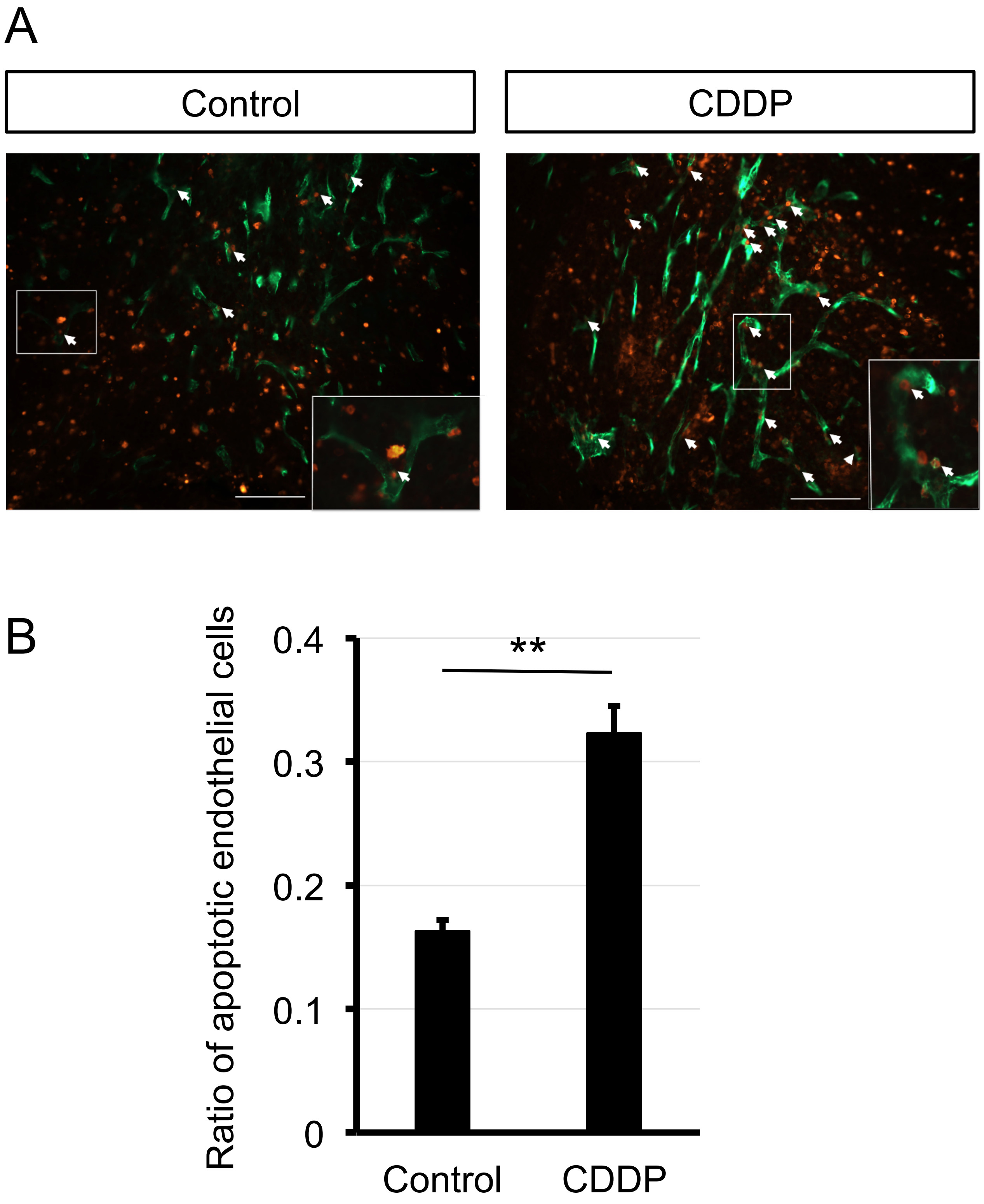

Supplementary Fig. 1. Apoptosis in the vasculature after CDDP treatment

Immunofluorescence analysis to detect apoptosis of endothelial cells in tumors. A: Sections of LLC tumors were double stained with anti-CD31 antibody (green) and anti-cleaved caspase-3 antibody (red). Left panel: control group, right panel: CDDP-treated group. Arrows point to cleaved caspase-3–positive nuclei in endothelial cells. Insets show higher magnification of the areas indicated in the boxes. Scale bar: 200 μm. B: Ratio of apoptotic endothelial cells among total endothelial cells. More than 3 random fields in each tumor were evaluated at a magnification of x100. Data show mean ± SEM; $^{**}p < 0.01$.
